# Effects of okadaic acid, azaspiracid-1, yessotoxin and their binary mixtures on human intestinal Caco-2 cells

**DOI:** 10.17179/excli2023-6884

**Published:** 2024-04-22

**Authors:** Jimmy Alarcan, Albert Braeuning

**Affiliations:** 1 German Federal Institute for Risk Assessment, Department of Food Safety, Max-Dohrn-Straße 8-10, 10589 Berlin, Germany

**Keywords:** phycotoxins, mixtures, inflammation, Caco-2 cells

## Abstract

Phycotoxins are responsible for foodborne intoxications. Symptoms depend on the ingested toxins but mostly imply gastro-intestinal and neurological disorders. Importantly, humans are exposed to combinations of several phycotoxins, resulting in possible mixture effects. Most previous studies, however, have been focused on single toxin effects. Thus, the aim of this study was to examine the effects of binary mixtures of three main phycotoxins, okadaic acid (OA), azaspiracid-1 (AZA1) and yessotoxin (YTX), on human intestinal Caco-2 cells. The focus was placed on cell viability studies and inflammation responses using a multi-parametric approach to assess cell population (nuclei staining), cell metabolism/viability (reductase activity and lysosomal integrity), and release of inflammation markers (e.g., interleukins). Mixture effects were evaluated using the concentration addition (CA) and independent action (IA) models. Our assays show that none of the toxins had an impact on the cell population in the tested concentration range. Only OA modulated reductase activity, while all three toxins had strong effects on lysosomal integrity. Furthermore, all toxins triggered the release of interleukin 8 (IL-8), with OA being most potent. Mixture effect analysis showed additivity in most cases. However, supra-additivity was observed in regards to IL-6 and IL-8 release for combinations implying high concentrations of OA. This study extends the knowledge on mixture effects of phycotoxins in human cells.

## Introduction

Phycotoxins are produced by phytoplankton and accumulate in seafood, especially in filtering species like clams, mussels or oysters (Visciano et al., 2016[32]). When humans consume contaminated seafood, this can lead to intoxications with different symptoms depending to the toxin(s) ingested. Okadaic acid (OA) is responsible for so-called diarrheic shellfish poisoning (DSP) characterized by symptoms like diarrhea, nausea, vomiting, abdominal cramps (Valdiglesias et al., 2013[31]). Azaspiracid-1 (AZA1) provokes both gastro-intestinal and neurological symptoms (Furey et al., 2010[17]). Even if no human intoxication has been yet reported with yessotoxin (YTX), studies in rodents have shown cardiotoxic effects (Ferreiro et al., 2016[13]; Tubaro et al., 2008[30]). To ensure safety to the consumers, regulatory thresholds have been established for toxins at the EU level (EFSA, 2009[12]). However, phycotoxins contamination mostly occurs as mixtures, and the issue of mixtures is not yet fully addressed in the field of phycotoxins (Alarcan et al., 2018[5]). In its opinion report, the European Food Safety Authority (EFSA) apprehends mixtures in the case of toxin analogs and describes toxicity equivalent factors (TEFs) that have been established (EFSA, 2009[12]). Only few studies regarding deleterious effects induced by mixtures of less closely related phycotoxins have been conducted, and it is noteworthy that deviations from additivity have been reported (Alarcan et al., 2018[5], 2019[4]). Therefore, the impact of mixtures of different groups of toxins needs to be investigated in more depths, as highlighted in the recommendations section of the abovementioned EFSA report.

The gastrointestinal epithelium acts as a mechanical barrier to limit the crossing and absorption of harmful substances. Thus, the integrity of the barrier is of key importance to prevent potential toxicity to systemic organs. Oral exposure to phycotoxins has shown to induce toxicity in rodents with macroscopical intestinal damage such as cell detachment, fluid accumulation, villous erosion and dilatation of the intestine tract (Aasen et al., 2010[1]; Aune et al., 2012[7]; Ito et al., 2000[19], 2002[18]). In addition, infiltration of immune cells in the lamina propria was observed, underlining potential inflammation of the gut (Aasen et al., 2011[2]; Aune et al., 2012[7]; Sosa et al., 2013[29]). At the cellular level, OA was reported to induce cytotoxicity, genotoxic effects and the release of IL-8 in human intestinal Caco-2 cells (Alarcan et al., 2019[4]). In the same study, YTX was not reported to induce any toxicity but interestingly, a binary mixture of YTX and OA produced higher levels of IL-8 release, suggesting some potentiation effect of YTX. In a co-culture of Caco-2 and HT29-MTX cells, both AZA1 and YTX were reported to induce weak cytotoxic effects without an induction of IL-8 release, while OA induced strong release of the interleukin (Reale et al., 2021[25]). The same group showed that all three toxins induced the translocation of NFκB in rat enteric glial cell (Reale et al., 2019[26]). The translocation of NFκB by OA was also reported in proliferative Caco-2 and HT29-MTX cells, as well as HepaRG cells (Ferron et al., 2014[15]; Wuerger et al., 2023[33]). NFκB plays a major role in inflammation as, upon activation, it induces the expression of various pro-inflammatory genes, especially genes encoding cytokines and chemokines (Liu et al., 2017[22]). Therefore, the pro-inflammatory potential of phycotoxins needs to be further elucidated and their possible effects in mixtures are also of key importance considering their documented co-exposure in seafood. 

Different mathematical models have been proposed to predict the combination effects of mixtures (Foucquier and Guedj, 2015[16]; Lasch et al., 2020[21]). Traditionally, models predict additivity, i.e., the combined effect that results from the contribution of each component inside the mixture, with the assumption that each component does not modify the effect of the other components. The two most recognized additivity models are concentration addition (CA), which applies for compounds with similar mechanisms of action, and independent action (IA) for compounds with dissimilar mechanisms of action (EFSA, 2009[12]). Owing to the diverse mechanisms of action of the chosen phycotoxins (OA is a potent PP2A/PP1 inhibitor, AZA1 interferes with ionic channels, and the exact mechanism of YTX is still unclear) and their CYP-mediated metabolism, some toxicokinetic interactions may take place, which could eventually result in non-additivity effects when the toxins are present in mixtures.

In this study, we investigated whether OA, AZA1 and YTX trigger toxicological responses in human intestinal Caco-2 cells, and how mixtures of toxins modulate these toxicities.

## Materials and Methods

### Chemicals

OA was purchased from Enzo Life Sciences GmbH (98 %, Lörrach, Germany). AZA1 and YTX were purchased from CIFGA (Lugo, Spain). Triton-X-100 was purchased from AppliChem (Darmstadt, Germany). Lipopolysaccharide (LPS) was purchased from Merck (Darmstadt, Germany). All other chemicals including ethanol, methanol (MeOH), and acetic acid were of analytical grade and purchased from ThermoFisher Scientific (Leicestershire, UK). Deionized water was prepared using a Milli-Q system (Millipore, Bedford, MA, USA). 

### Cell culture 

Caco-2 cells were obtained from the European Collection of Cell Cultures (Salisbury, UK). Cells (passages 30-38) were seeded at 10,000 cells/cm^2^ in 96-well plates and cultured in Dulbecco's Modified Eagle's Medium (DMEM) high glucose (PAN-Biotech GmbH, Aidenbach, Germany) supplemented with 10 % fetal bovine serum (FBS), 100 U/mL penicillin and 100 μg/mL streptomycin (all from Capricorn Scientific, Ebsdorfergrund, Germany). Cells were maintained at 37 °C in a humidified atmosphere at 5 % CO_2_. For differentiation into an intestinal epithelial-like monolayer, cells were cultured for 3 weeks with renewal of medium every 2 to 3 days. Treatment with toxins was performed in serum-free DMEM medium (without phenol red) to avoid interaction of compounds with serum components.

### Mixture design

Three binary mixtures were designed with the following molar ratios: 3:1 for OA/AZA1 and OA/YTX and 1:1 for AZA1/YTX. Ratios were selected on the basis of published literature on toxin co-exposure (Alarcan et al., 2018[5]). The maximal test concentration for OA was chosen based on published data and further concentrations for AZA1 and YTX were deduced based on the ratios of toxins as previously indicated (Table 1(Tab. 1)).

### Cell viability 

#### Cell count - Hoechst 33342

After 24 h of treatment with toxins, the culture medium was removed and PBS containing Hoechst 33342 solution (5 µg/ml) was added for 10 min at 37 °C. Fluorescence was measured at λ_exc_ = 350 nm and λ_em_ = 461 nm using an Infinite 200 Pro microplate reader (Tecan, Männedorf, Switzerland).

#### Reductase activity - CTB assay

After 24 h of treatment with toxins, cell reductase activity was measured by using the CellTiter-Blue Cell Viability Assay (Promega, Madison, WI, USA). CTB reagent (diluted 1:4 in PBS) was directly added to the cells for 2 h at 37 °C. Fluorescence was measured at λ_exc_ = 560 nm and λ_em_ = 590 nm using an Infinite 200 Pro microplate reader (Tecan, Männedorf, Switzerland).

#### Lysosomal integrity - Neutral red uptake 

After 24 h of treatment with toxins, the culture medium was removed and cells were washed with PBS. Neutral red solution (40 µg/ml) was added to the cells and incubated for 2 h at 37 °C. Neutral red solution was thereafter removed and solubilization solution (50 % ethanol, 49 % H_2_O, 1 % glacial acetic acid) was added to the cells. Plates were put for 30 min on a plate shaker at room temperature until total solubilization. Fluorescence was measured at λ_exc_ = 530 nm and λ_em_ = 645 nm using an Infinite 200 Pro microplate reader (Tecan, Männedorf, Switzerland). 

#### Cell membrane damage - Lactate dehydrogenase activity 

After 24 h of treatment with toxins, cell supernatants were transferred to 96-well microplates. Reaction mixture solution (Cytotoxicity Detection Kit, Roche, Mannheim, Germany) was freshly prepared and added to the samples. Plates were incubated for 30 min at room temperature. Absorbance was measured at 491 nm with a reference wavelength of 650 nm using an Infinite 200 Pro microplate reader (Tecan, Männedorf, Switzerland).

### Beads-Luminex assay

Levels of IL-1β, IL-6, IL-8, S100A8/A9, and galectin-3 were determined using Human ProcartaPlex Multiplex kits (ThermoFisher Scientific) following the instructions provided by the manufacturer. Briefly, following 24 h treatment with toxins or the positive control LPS (10 ng/ml), cell supernatants were transferred to 96-well microplates and stored at -80 °C until further analysis. After accumulation of the magnetic bead solution to 96-well flat bottom plate, samples were added and incubated overnight. Following washing steps, detection antibody mix was incubated for 30 minutes on a plate shaker at 500 rpm. After washing, streptavidin peroxidase was added for 30 min on a plate shaker at 500 rpm. After a final washing step, reading buffer was added and the plate was analyzed using a Bio-Plex 200 Systems reader (Bio-Rad).

### Statistics/Data analysis

GraphPad Prism 9 (GraphPad Software, Inc) was used for statistical analyses. One-way ANOVA (analysis of variance) followed by Dunnett's post-hoc test was used to compare the effects of chemicals and solvent control. Symbols *, **, and *** indicate statistical significance between toxin and solvent control (p < 0.05, p < 0.01, and p < 0.001, respectively).

### Mixture effect predictions 

#### Concentration addition method

The CA method was used as described by Lasch et al. (2020[21]). This method is used when compounds have similar mechanisms of action. In our study of binary phycotoxin combinations, the predicted mixture effect values E_mix_ were calculated as follows: 

*E_mix_* = (*p_A_/E_A_* + *p_B_/E_B_*)^-1^

with E_A_ and E_B_ denoting the effect of compound A and B, while p_A_ and p_B_ denote the fraction of compound A and B in the mixture, i.e., the concentration of compound alone versus the total concentration of the mixture.

#### Independent action concept

The IA concept was used as described by Lasch et al. (2020[21]). In our study, for binary phycotoxin combinations, the predicted mixture effect values E_mix_ were calculated as follows: 

*E_mix_* = *E_A_* + *E_B _*- (*E_A_* ⨯ *E_B_*)

with E_A_ and E_B_ denoting the effect of compound A and B.

To establish the predictions for endpoints other than cell viability, the raw data originally expressed as fold change compared to the respective solvent control were processed to obtain data suitable for IA analysis. Thus, data were converted to values between 0 and 1. For each endpoint, the condition among the chemicals tested individually and the binary mixture showing the highest effect was attributed the value of 1 (A_max_). All other conditions were then normalized to the A_max_. This data processing was described previously by Alassane-Kpembi et al. (2017[6]) and allows assessing combination effects for endpoints other than cell viability. The raw data from cell viability assays were divided by 100 to get values between 0 and 1. Cell viability values superior to 100 % relative viability were set to 1.

#### Comparison of the models and thresholds of additivity

Combination index (CI) and model deviation ratio (MDR) are both standard indicators of combination effects and were calculated as indicated by Lasch et al. (2020[21]): 

*CI* = *MDR* = predicted value for the mixture / measured value for the mixture

The CI approach provides conservative thresholds: CI< 0.9, 0.9 ≤ CI ≤ 1.1, and CI > 1.1 were set to indicate synergism, additive effects and antagonism, respectively (Chou, 2006[10]). The MDR approach provides larger thresholds to avoid possible misinterpretation: MDR< 0.5, 0.5 ≤ MDR ≤ 2, and MDR > 2 were set to indicate synergism, additive effects and antagonism, respectively (Belden et al. 2007[8]).

## Results

### Toxic effects following OA/AZA1 mixtures treatment in Caco-2 cells 

**Figure 1:**
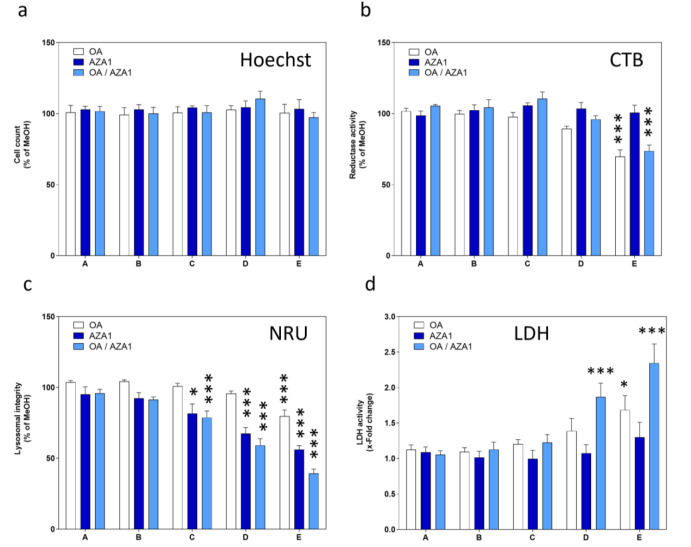
Effects of OA/AZA1 mixtures on a panel of toxicity endpoints in differentiated Caco-2 cells. Cells were incubated with the toxins for 24 h before measurement of (a) cell count, (b) reductase activity, (c) lysosomal integrity, (d) LDH activity. Triton-X-100 (0.05 %) was used as positive control for all four endpoints. Results were obtained from four independent experiments, each performed in triplicates. Data represents means and SEM of fold change compared to solvent control. *, **, *** indicate statistical significance between toxin and solvent control (p < 0.05, p < 0.01, p < 0.001 respectively) after one-way ANOVA followed by Dunnett's post-hoc test.

The toxins had no effects on cell count in the chosen concentration range, neither alone nor in mixtures (Figure 1a(Fig. 1)). While AZA1 had no effect on reductase activity, OA induced a concentration-dependent decrease (Figure 1b(Fig. 1)). Mixtures of OA and AZA1 showed a similar response as for OA alone. All treatment conditions induced a concentration-dependent decrease in the lysosomal integrity (Figure 1c(Fig. 1)), with AZA1 being more potent than OA. OA induced a concentration-dependent increase in LDH activity (Figure 1d(Fig. 1)). Mixtures of OA and AZA1 led to a more pronounced response than OA alone for the two highest concentrations.

**Figure 2:**
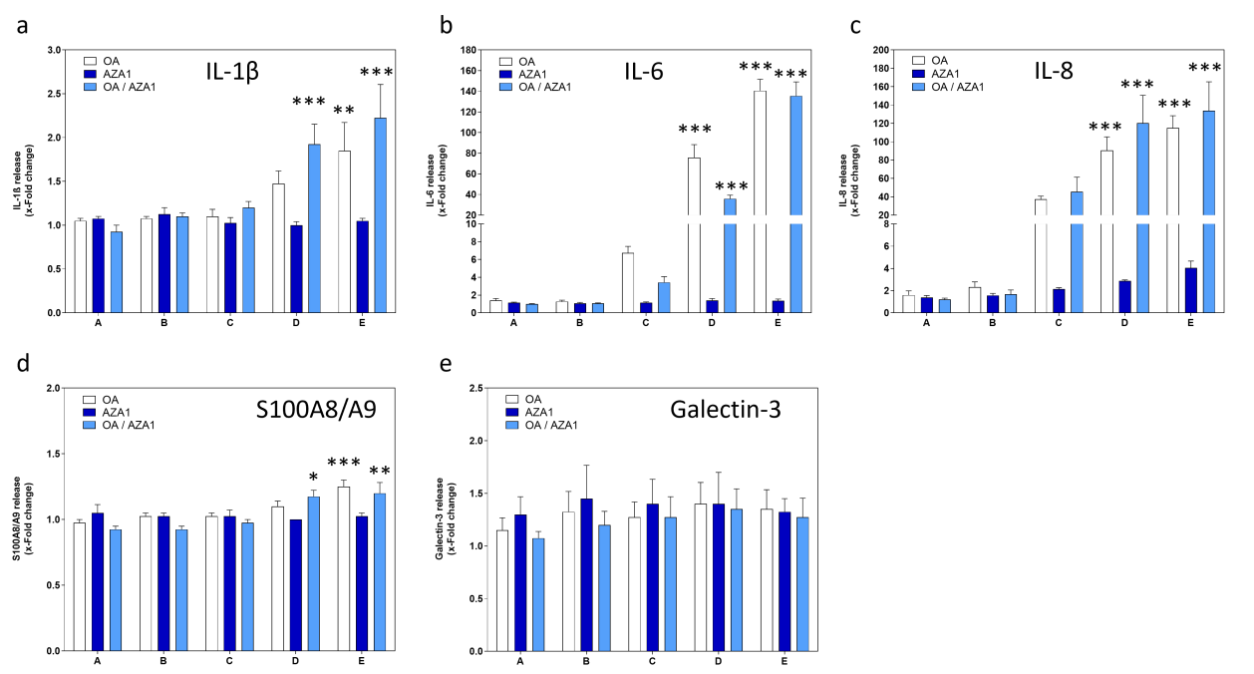
Effects of OA/AZA1 mixtures on a panel of inflammation markers in differentiated Caco-2 cells. Cells were incubated with the toxins for 24 h. Cell supernatants were collected and analyzed for (a) IL-1β, (b) IL-6, (c) IL-8, (d) S100A8/A9, (e) Galectin-3. LPS (10 ng/ml) was used as positive control for all endpoints. Results were obtained from four independent experiments, each performed in triplicates. Data represents means and SEM of fold change compared to solvent control. *, **, *** indicate statistical significance between toxin and solvent control (p < 0.05, p < 0.01, p < 0.001 respectively) after one-way ANOVA followed by Dunnett's post-hoc test.

OA induced a concentration-dependent increase in IL-1β, IL-6 and IL-8 in cell culture supernatants, while AZA1 only induced the release of IL-8 (Figure 2a to c(Fig. 2)). Similar outcomes as for OA alone were observed for mixtures of OA and AZA1. Very slight increases in S100A8/A9 were observed at high concentrations for OA alone and in mixture with AZA1 (Figure 2d(Fig. 2)). The two toxins, neither alone nor in mixtures, had effects on galectin-3 (Figure 2e(Fig. 2)). LPS only had an effect on IL-8 release (2.8-fold induction).

### Toxic effects following OA/YTX mixtures treatment in Caco-2 cells 

**Figure 3:**
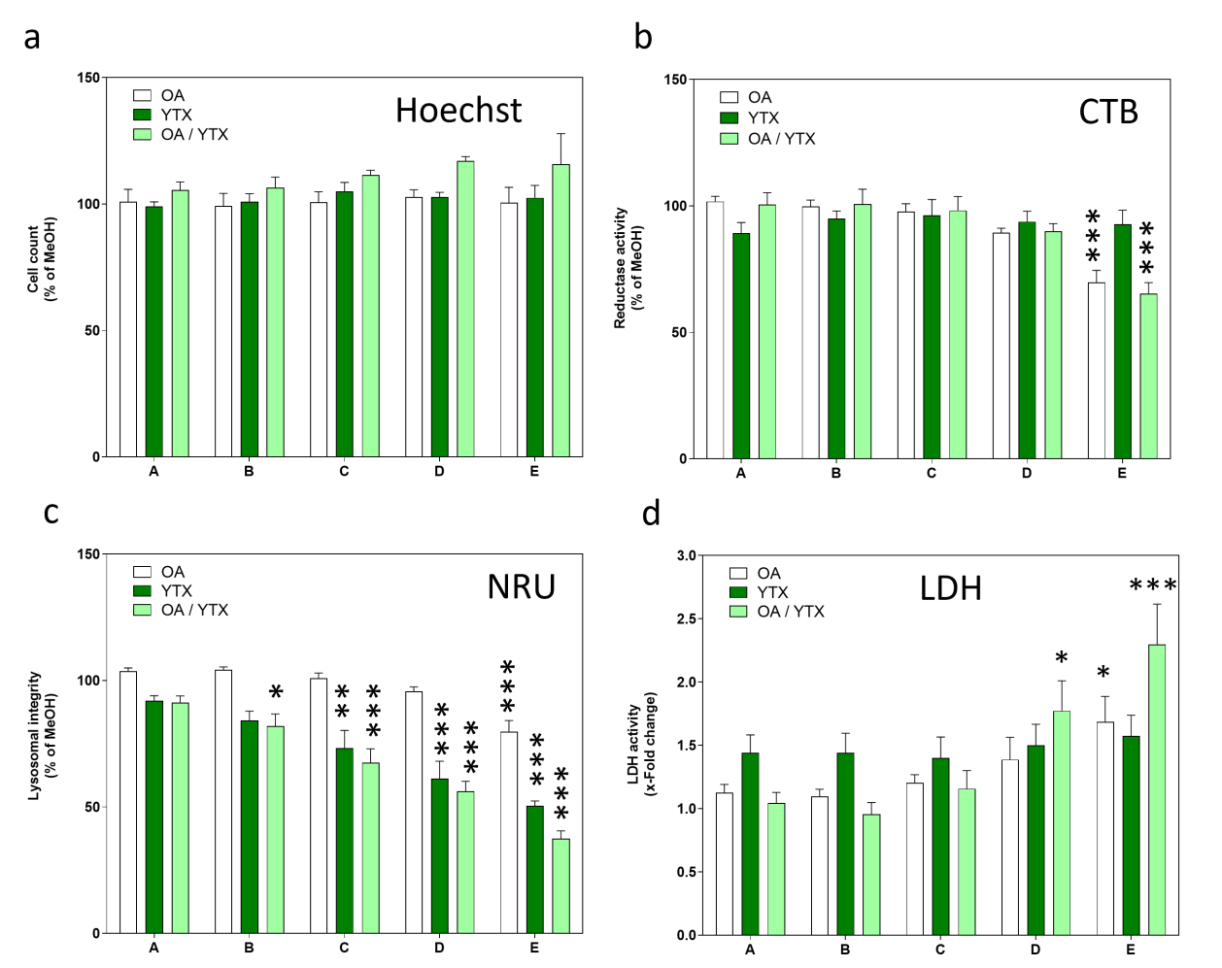
Effects of OA/YTX mixtures on a panel of toxicity endpoints in differentiated Caco-2 cells. Cells were incubated with the toxins for 24 h before measurement of (a) cell count, (b) reductase activity, (c) lysosomal integrity, (d) LDH activity. Triton-X-100 (0.05 %) was used as positive control for all four endpoints. Results were obtained from four independent experiments, each performed in triplicates. Data represents means and SEM of fold change compared to solvent control. *, **, *** indicate statistical significance between toxin and solvent control (p < 0.05, p < 0.01, p < 0.001 respectively) after one-way ANOVA followed by Dunnett's post-hoc test.

The toxins had no effects on cell count in the chosen concentration range, neither alone nor in mixtures (Figure 3a(Fig. 3)). Regarding reductase activity, while YTX had no effect, OA induced a concentration-dependent decrease (Figure 3b(Fig. 3)). Mixtures of OA and YTX showed a similar response as for OA alone. All treatment conditions induced a concentration-dependent decrease in the lysosomal integrity (Figure 3c(Fig. 3)), with YTX being more potent than OA. OA induced a concentration-dependent increase in LDH activity (Figure 3d(Fig. 3)). Mixtures of OA and YTX led to a more pronounced response than OA alone for the two highest concentrations.

**Figure 4:**
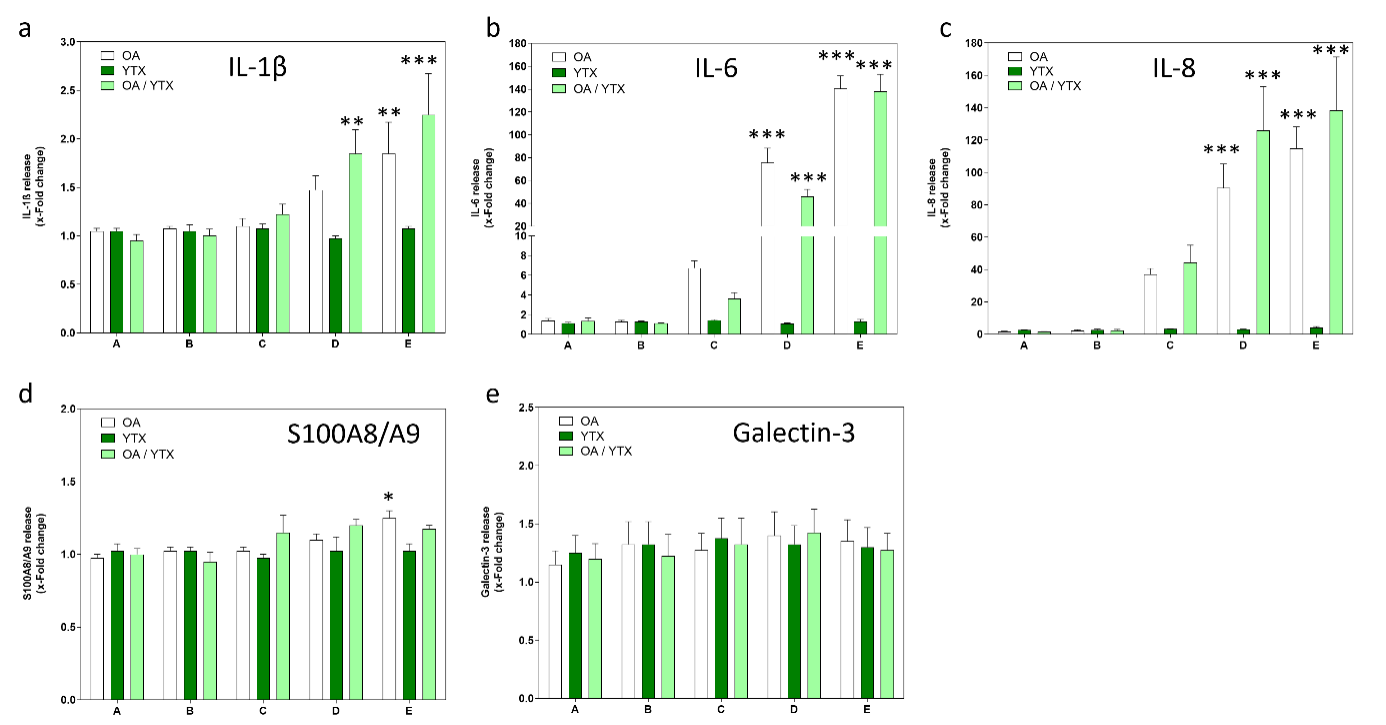
Effects of OA/YTX mixtures on a panel of inflammation markers in differentiated Caco-2 cells. Cells were incubated with the toxins for 24 h. Cell supernatants were collected and analyzed for (a) IL-1β, (b) IL-6, (c) IL-8, (d) S100A8/A9, (e) Galectin-3. LPS (10 ng/ml) was used as positive control for all endpoints. Results were obtained from four independent experiments, each performed in triplicates. Data represents means and SEM of fold change compared to solvent control. *, **, *** indicate statistical significance between toxin and solvent control (p < 0.05, p < 0.01, p < 0.001 respectively) after one-way ANOVA followed by Dunnett's post-hoc test.

OA induced a concentration-dependent increase in IL-1β, IL-6 and IL-8 in cell culture supernatants, while YTX only induced the release of IL-8 (Figure 4a to c(Fig. 4)). Similar outcomes as for OA alone were observed for mixtures of OA and YTX. Very slight increases in S100A8/A9 were observed at high concentrations for OA alone (Figure 4d(Fig. 4)). The two toxins, neither alone nor in mixtures, had effects on galectin-3 (Figure 4e(Fig. 4)). LPS only had an effect on IL-8 release (2.8-fold induction).

### Toxic effects following AZA1/YTX mixtures treatment in Caco-2 cells 

**Figure 5:**
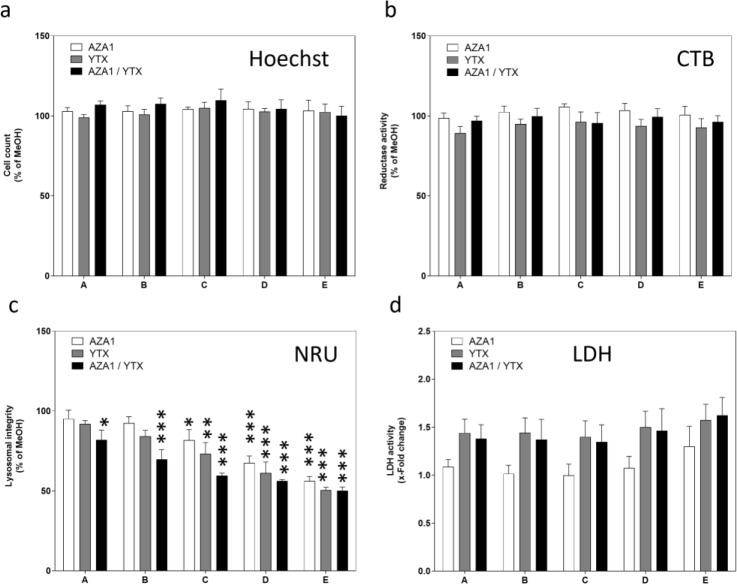
Effects of AZA1/YTX mixtures on a panel of toxicity endpoints in differentiated Caco-2 cells. Cells were incubated with the toxins for 24 h before measurement of (a) cell count, (b) reductase activity, (c) lysosomal integrity, (d) LDH activity. Triton-X-100 (0.05 %) was used as positive control for all four endpoints. Results were obtained from four independent experiments, each performed in triplicates. Data represents means and SEM of fold change compared to solvent control. *, **, *** indicate statistical significance between toxin and solvent control (p < 0.05, p < 0.01, p < 0.001 respectively) after one-way ANOVA followed by Dunnett's post-hoc test.

The toxins had no effects on cell count and reductase activity in the chosen concentration range, neither alone nor in mixtures (Figure 5a and b(Fig. 5)). All treatment conditions induced a concentration-dependent decrease in the lysosomal integrity (Figure 5c(Fig. 5)), with YTX being slightly more potent than AZA1. YTX induced an increase in LDH activity albeit without statistical significance (Figure 5d(Fig. 5)). A similar response as for YTX alone was observed for mixtures of AZA1 and YTX.

**Figure 6:**
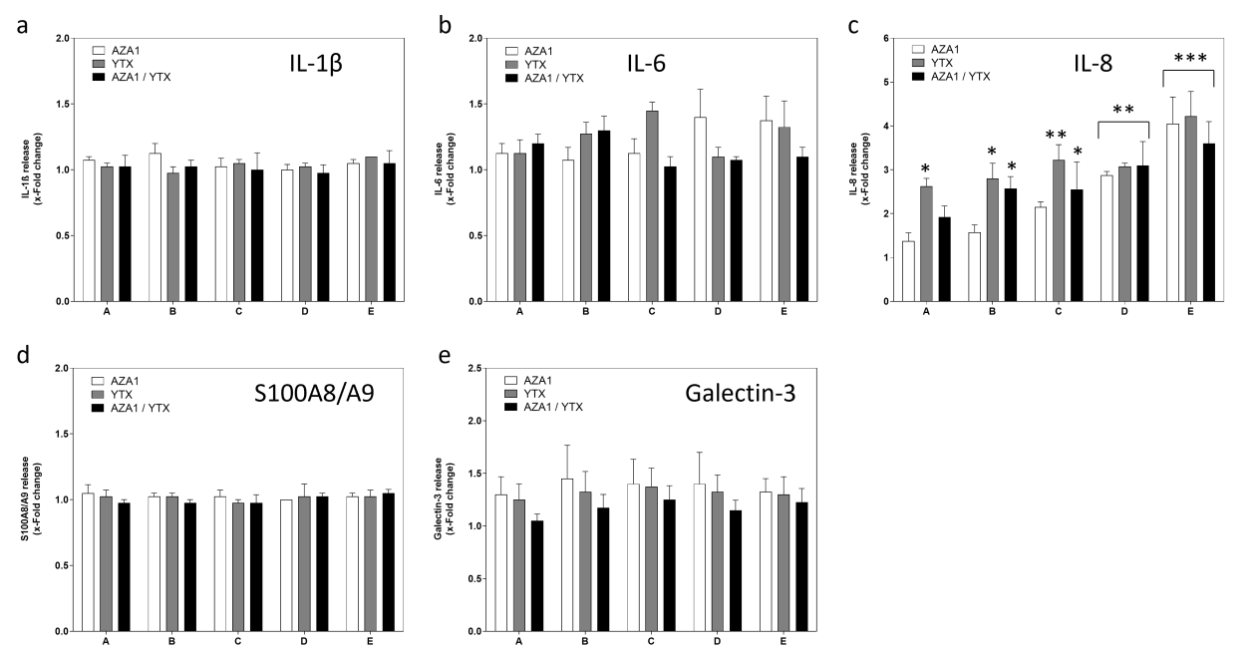
Effects of AZA1/YTX mixtures on a panel of inflammation markers in differentiated Caco-2 cells. Cells were incubated with the toxins for 24 h. Cell supernatants were collected and analyzed for (a) IL-1β, (b) IL-6, (c) IL-8, (d) S100A8/A9, (e) Galectin-3. LPS (10 ng/ml) was used as positive control for all endpoints. Results were obtained from four independent experiments, each performed in triplicates. Data represents means and SEM of fold change compared to solvent control. *, **, *** indicate statistical significance between toxin and solvent control (p < 0.05, p < 0.01, p < 0.001 respectively) after one-way ANOVA followed by Dunnett's post-hoc test.

The toxins had no effects on IL-1β, IL-6, S100A8/A9, and galectin-3 in the chosen concentration range, neither alone nor in mixtures (Figure 6a, b, d, and e(Fig. 6)). All treatment conditions induced a concentration-dependent increase in IL-8 (Figure 6c(Fig. 6)), with YTX being slightly more potent than AZA1. LPS only had an effect on IL-8 release (2.8-fold induction).

### Predictions of mixture effects using CA and IA 

**Figure 7:**
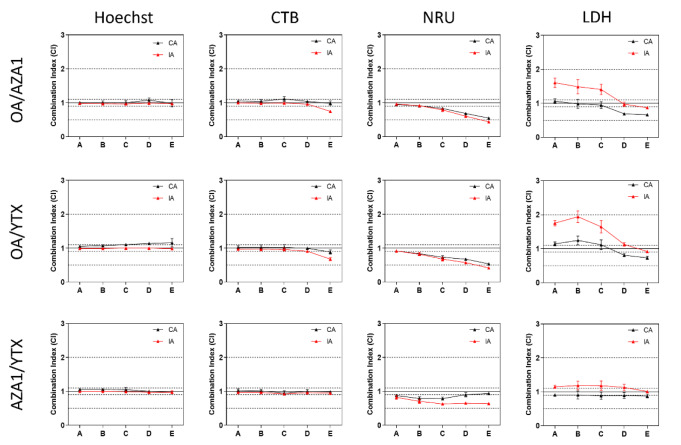
Analysis of mixtures using CA and IA on a panel of toxicity endpoints in differentiated Caco-2 cells. CI < 0.9, 0.9 ≤ CI ≤ 1.1 and CI > 1.1 indicate respectively synergism, additivity and antagonism. MDR< 0.5, 0.5 ≤ MDR ≤ 2, and MDR > 2 were set to indicate synergism, additivity and antagonism, respectively. Data represents means and SEM from four independent experiments, each performed in triplicates. Dashed lines indicate lower and upper limits of additivity according to CI or MDR.

According to the MDR thresholds, no deviations from additivity were observed with respect to the cell viability-related parameters cell count, reductase activity, lysosomal integrity, and LDH activity (Figure 7(Fig. 7)). This was observed for all binary mixtures, with the sole exception of the endpoint lysosomal integrity for the highest concentrations of the mixtures OA/AZA1 and OA/YTX, where supra-additivity (based on the prediction from IA but not from CA) was observed (Figure 7(Fig. 7)). When applying the CI thresholds, many more deviations from additivity can be pointed out. For instance, all three binary mixtures showed increasing synergism towards lysosomal integrity as the test concentrations increase. At low concentrations, the mixture of OA and YTX showed a deviation in the way of antagonism in regards to LDH activity.

**Figure 8:**
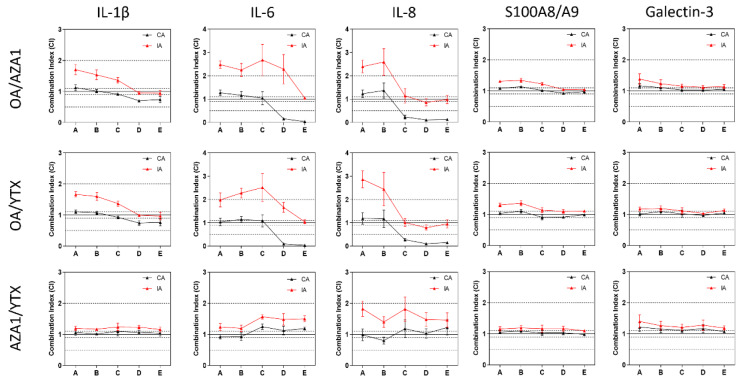
Analysis of mixtures using CA and IA on a panel of inflammation markers in differentiated Caco-2 cells. CI < 0.9, 0.9 ≤ CI ≤ 1.1 and CI > 1.1 indicate respectively synergism, additivity and antagonism. MDR< 0.5, 0.5 ≤ MDR ≤ 2, and MDR > 2 were set to indicate synergism, additivity and antagonism, respectively. Data represents means and SEM from four independent experiments, each performed in triplicates. Dashed lines indicate lower and upper limits of additivity according to CI or MDR.

According to MDR thresholds, no deviations from additivity were observed towards IL-1β, S100A8/A9 or galectin-3 release, irrespective of the mathematical model (Figure 8(Fig. 8)). However, based on the prediction from the IA model, deviations in the way of antagonism were observed for OA/AZA-1 and OA/YTX in regards to IL-6 and IL-8 release (Figure 8(Fig. 8)). On the contrary, based on the predictions from the CA model, deviations in the way of synergism were observed for the highest concentrations. When applying the CI thresholds, additional deviations from additivity can be pointed out. For instance, OA/AZA-1 and OA/YTX mixtures showed synergism towards IL-1β release at high concentrations. At low concentrations, deviation in the way of antagonism were observed based on the IA predictions.

## Discussion

In this work, we aimed at studying the possible toxic effects of binary mixtures of lipophilic phycotoxins in an *in vitro* model of the human intestine. We designed our mixtures according to a literature review on the occurrence of phycotoxin mixtures (Alarcan et al., 2018[5]). Owing to the complexity in the contamination profile of seafood, we restricted the mixtures to binary combinations. Thus, three mixtures were tested, involving the main toxins OA, AZA1 and YTX, and reflecting documented exposure scenarios. The scope of the work was to determine cytotoxicity via different, independent assays, and quantify a possible inflammation-related response of the Caco-2 cells when exposed to the single compounds or mixtures.

Our results highlight the importance of selecting several cytotoxicity assays targeting different cell compartments and/or metabolic processes. Most metabolic assays (e.g., the MTT assay, or assays to measure cellular ATP levels) are being used as a way to assess cell survival (i.e., as a surrogate for the cell number). The CTB assay used in this study measures the activity of mitochondrial, but also cytosolic dehydrogenases. Its results might thus, at least to a certain degree, also reflect possible interference of the test compounds with mitochondrial function. Our data shows that metabolic assays can reveal a disturbance of metabolic homeostasis, without the cell population being affected. AZA1 and YTX did not induce any decrease in the cell population and reductase activity, while they drastically impaired lysosomal function, as documented by the results of the neutral red uptake (NRU) assay. The cell viability assays that are traditionally used to determine, for example, the appropriate concentration range of chemicals to use for further assays, can thus, in specific cases, provide some indication of the mode of action of the tested substance. In regards to our results, it can be hypothesized that AZA1 and YTX specifically interfere with the lysosomal pathway to exert their toxicity. Accordingly, effects of YTX on lysosomal function and more globally on autophagy have been documented in skeletal BC3H1 cells and fibroblast NIH3T3 cells (Korsnes et al., 2016[20]; Malagoli et al., 2006[23]). Moreover, AZA was shown to induce autophagosomes inside the cytoplasm of Caco-2 cells (Abal et al., 2017[3]). The exact mechanism of action is still to be characterized for both AZA1 and YTX, but considering the principle of the NRU assay (i.e., the pH-dependent incorporation of the dye into lysosomes), it can be speculated that both toxins disrupt cellular or at least lysosomal pH homeostasis. Mechanisms behind pH modulation are multiple and may involve, for instance, modulation of Ca^2+^ levels or ATP production. More specific investigation of mitochondrial function in future studies might be useful to further characterize the cytotoxicity profile of marine biotoxins. Of note, this should be done with caution, as cytotoxicity assays specifically targeting mitochondrial functions may yield misleading results at least for AZA, since it has been shown in liver cells that the toxin leads to increased mitochondrial dehydrogenase activities (Pelin et al., 2019[24]).

Our study revealed that OA did not reduce the overall cell population in the concentration range studied, but disrupted cellular metabolism as shown by the decrease in reductase activities and lysosomal activities. These effects were accompanied by an induction of IL-1β, IL-6, and IL-8 release. The induction of interleukins points toward a pro-inflammatory response triggered by OA. This is in line with our previous work where we observed induction of IL-8 following OA treatment in Caco-2 cells (Alarcan et al., 2019[4]). The induction of IL-6 and IL-8 release was also observed in human HepaRG cells (Wuerger et al., 2023[33]). As opposed to the strong interleukin induction, no effects were observed in regards to galectin-3 and S100A8/A9. Interleukins are under the control of NFκB, while galectin-3 and S100A8/A9 are under the control of the transcription factors AP-1 and PU.1, respectively (Liu et al., 2017[22]; Song et al., 2005[28]; Xu et al., 2021[34]). Thus, it can be hypothesized that OA only activated NFκB to induce its pro-inflammatory effects. Translocation of NFκB following OA treatment in glial cells, HepaRG cells and proliferating Caco-2 cells support this hypothesis (Ferron et al., 2014[15]; Reale et al., 2019[26]; Wuerger et al., 2023[33]). Further downstream, activation of JAK/STAT was shown in human HepaRG liver cells (Wuerger et al., 2023[33]), but current data in this study do not permit to confirm that similar signaling takes place in Caco-2 cells. Such mechanistic characterization is, however, beyond the scope of this study. AZA1 and YTX had no effects on inflammation markers except for IL-8, where slight increases were observed at high concentrations (4-fold, as opposed to 40-fold increases observed for OA). In previous work with co-cultures of Caco-2 and HT29-MTX cells, neither AZA1 nor YTX were reported to induce IL-8 release (Reale et al., 2021[25]). However, both toxins induced the translocation of NFκB and the release of the inflammation markers S100β and iNOS in rat enteric glial cells (Reale et al., 2019[26]). Thus, the potential for pro-inflammatory effects of AZA1 and YTX may be related to their uptake and/or metabolism.

We used the two main existing additivity models to assess the combination effects of toxins. The use of multiple models has been advised by many studies and helps to increase the confidence in the evaluation of mixture effects (Foucquier and Guedj, 2015[16]; Lasch et al., 2020[21]; Zhao et al., 2010[35]). Moreover, we used the two main additivity thresholds (i.e., CI and MDR) to evaluate the data for possible deviations. If MDR use is recommended to prevent false mixture effect allegations, it is noteworthy that in the case of an endpoint with low maximum signal response (for instance 1.8-2-fold), it gets virtually impossible to observe a deviation from additivity in the way of synergism using such thresholds. On the contrary, in the case of an endpoint with high maximum signal response (for instance 50-fold), the use of CI thresholds may result in erroneous synergism claims. Thus, we would advocate to take into account considerations of the maximum signal and signal dynamics for a wise use of CI and MDR thresholds.

We show that additivity was correctly predicted irrespective of the mixture for the tested endpoints cell count and reductase activity. This indicates that the toxins do not interact and do not modulate their toxic effects in regards to those endpoints. The fact that the CA and IA models reached similar predictions while being designed for different biological situations does not raise concern, as such outcome has been observed multiple times (see the review by Cedergreen et al. (2008[9])). Regarding lysosomal activity, deviations from additivity were observed in the direction of stronger effects with mixtures involving OA. A similar outcome was observed for IL-1β, IL-6 and IL-8 release with CA modeling (with CI thresholds for IL-1β). It is noteworthy that AZA1 and YTX alone showed no effect towards IL-1β and IL-6, meaning that they may potentiate the OA response. The deviation from additivity in the case of IL-8 release was previously observed in Caco-2 cells with mixtures of OA and YTX (Alarcan et al., 2019[4]). This indicates a potential for synergistic interactions between OA and AZA1 or YTX. It is difficult to elaborate on a possible toxicodynamic mechanism that occurs in mixture as OA, being a potent PP2A inhibitor, interferes with a myriad of cellular pathways. However, knowing that OA has been shown to induce the translocation of NFκB in different cell lines, an interaction with this specific transcription factor can be hypothesized. To support this view, some studies have shown the formation of so-called supramolecular ligands within the ligand-binding pocket of PXR or PPARα, leading to synergistic effect of the investigated mixtures (Delfosse et al., 2015[11]; Soderstrom et al., 2022[27]). The conclusion for the AZA1/YTX mixture is less clear, as different outcomes were observed depending on the model. Additivity or very slight synergism was observed with the CA model, which is consistent with the data reported by Ferron et al. (2016[14]) on proliferative Caco-2 cells with an almost equimolar mixture of AZA1 and YTX. On the other side, deviations from additivity predicted by the IA model were observed, which would indicate synergistic interaction. Conflicting outcomes make it difficult to draw a clear conclusion and further study is needed to confirm or infirm the possible synergistic effect of AZA1 and YTX.

In this study, we examined the toxicity of binary mixtures of phycotoxins on human intestinal Caco-2 cells. Mainly additive effects were observed but, in the case of IL-1β, IL-6 and IL-8, crescent synergism was reported with increasing concentrations. The mechanisms involved in the synergistic effects require further investigation. We are aware that the results obtained *in vitro* in Caco-2 cells might not exactly reflect the behavior of intestinal cells *in vivo* or of other cell lines. Our study points out that more data on hazard assessment of lipophilic phycotoxins mixtures as well as on co-exposure conditions are required to ensure that the current toxin limits in shellfish are adequately sufficient to protect consumers in case of co-exposure.

## Declaration

### Authorship contributions

Participated in research design: Alarcan, Braeuning

Conducted experiments: Alarcan

Performed data analysis: Alarcan

Wrote or contributed to the writing of the manuscript: Alarcan, Braeuning

### Acknowledgments

This work was supported by the German Federal Institute for Risk Assessment (Grant no. 1322-746).

### Conflict of interest

The authors declare that they have no conflict of interest. 

## Figures and Tables

**Table 1:**
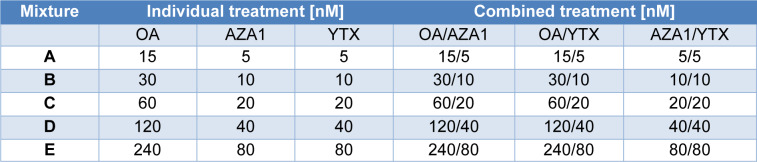
Design of binary mixtures
